# An Improved FBPN-Based Detection Network for Vehicles in Aerial Images †

**DOI:** 10.3390/s20174709

**Published:** 2020-08-20

**Authors:** Bin Wang, Yinjuan Gu

**Affiliations:** 1 Shanghai Institute for Advanced Communication and Data Science, Shanghai University, Shanghai 200444, China; brantley.wang@hotmail.com; 2 School of Communication and Information Engineering, Shanghai University, Shanghai 200444, China

**Keywords:** vehicle detection, aerial images, Feature Balanced Pyramid Network

## Abstract

With the development of artificial intelligence and big data analytics, an increasing number of researchers have tried to use deep-learning technology to train neural networks and achieved great success in the field of vehicle detection. However, as a special domain of object detection, vehicle detection in aerial images still has made limited progress because of low resolution, complex backgrounds and rotating objects. In this paper, an improved feature-balanced pyramid network (FBPN) has been proposed to enhance the network’s ability to detect small objects. By combining FBPN with modified faster region convolutional neural network (faster-RCNN), a vehicle detection framework for aerial images is proposed. The focal loss function is adopted in the proposed framework to reduce the imbalance between easy and hard samples. The experimental results based on the VEDIA, USCAS-AOD, and DOTA datasets show that the proposed framework outperforms other state-of-the-art vehicle detection algorithms for aerial images.

## 1. Introduction

Object detection has been a fundamental problem in computer vision. It plays an important role in various fields such as civil and security [1]. The development of object detection algorithms in the past 10 years can be roughly divided into two stages [2]. Before 2013, most algorithms rely on the hand-crafted features; after that, the algorithms are mainly based on CNN features. The traditional detection method can be summarized as three steps: “Region Selection”, “Feature Extraction” and “Classification”. “Region Selection” is a coarse locating process of the target. Since the targets may appear anywhere in the image and the sizes of the targets are uncertain, a sliding-window strategy [3] is used to traverse the image. To detect objects in different sizes, different scales and ratios are set for the sliding windows. Although the sliding-window strategy can obtain a large number of candidate regions, it also generates many redundant windows and the time complexity of this method is also high. “Feature Extraction” analyzes the candidate regions obtained in the previous step. Due to the background diversity, illumination changes, object occlusions, etc., it is not easy to design a feature with decent robustness. Because of the lack of effective image feature representation before deep learning, people have to design more diversified detection algorithms (including SIFT detection algorithm, histogram of gradients (HOG) detection algorithm and the DPM model [4,5,6] to compensate the defects of hand-crafted feature expression. “Classification” uses the region classifiers to assign categorical labels to the covered regions. Commonly, support vector machines are used here due to their good performance on small scale training data. In addition, some classification techniques such as bagging, cascade learning and Adaboost are used in region classification step, leading to further improvements in detection accuracy.

Although object detection methods based on traditional manual features are mature, they still face the following two problems: first, the region selection strategy based on a sliding window makes it easy to generate window redundancy and is time-consuming; second, hand-crafted features are not robust enough for the problem of object diversity. With the rapid development of deep-learning techniques, object detection algorithms based on deep learning have taken an important place. They can be classified into two major categories: one-stage methods and two-stage methods [3]. The methods which consist of three steps (candidate region proposal, feature extraction and classification) are well known as two-stage methods, such as the series of methods based on region convolutional neural network (RCNN [7], fast-RCNN [8], faster-RCNN [9], and feature pyramid networks [10]. In contrast, the methods which do not need any additional operation for region proposal, such as the YOLO series [11], SSD [12] and Retina-Net [3], are one-stage methods.

Small object detection is a branch of object detection, which is important for various applications, e.g., traffic management, urban planning, parking lot utilization, etc. Detection of ground vehicles or pedestrians by an unmanned aerial vehicle (UAV) and detection of ground objects by remote sensing images have been intensively explored by relevant researchers. Definition of a small object is usually different depending on specific applications. Bell S et al. proposes an inside-outside net (ION) structure and defines the small object as a target with a size of 32 × 32 pixels or less in a 1024 × 1024 image (COCO dataset [13]). While Maenpaa T et al. defines the small object with a size of approximately 20 × 20 pixels in a 512 × 512 image [14].

In this paper, we focus on vehicle detection in aerial images and propose a feature-balanced pyramid network (FBPN) for better feature extraction. The main contributions of this paper are presented as follows: (1) a specialized framework which combines FBPN with faster RCNN is proposed and applied to vehicle detection in aerial images. (2) An annotation method is designed to be more suitable for the proposed framework. (3) Data enhancement is proved to be effective in our proposed network.

## 2. Related Work

Prior to the development of deep learning, a sliding window detector [8] was widely used in object detection. Sliding window methods utilize both specific hand-crafted feature representations such as HOG and classifiers such as a support vector machine (SVM) to independently binary classify all sub-windows of an image as belonging to an object or background [15,16]. Even though their methods have made some improvements, hand-crafted features are insufficient to separate vehicles from complex background. Compared with sliding window methods, region proposal [9] can determine the location where the target may appear in the image in advance, which can reduce the computational overhead and improve the quality of candidate region. The series of methods based on region convolutional neural network (RCNN) uses region proposal for object detection and the results prove that they perform well when dealing with object detection tasks.

RCNN was proposed by Girshick et al. in 2014. This algorithm has three main steps. First, it extracts the object proposals in the image. Then, the proposals are adjusted to the same size and the features are extracted using the Alexnet network trained on ImageNet dataset. Finally, it uses the SVM classifier for false alarm elimination and category judgment. RCNN achieved good results on the VOC07 dataset, with mAP increasing from 33.7% (DPM-v5 [17]) to 58.5%. Although R-CNN has made great progress, its defects are also obvious. First, the training process of RCNN is multi-stage, which is cumbersome and time-consuming. Second, due to repeated feature extraction on high-density candidate regions, its detection speed is relatively slow (40 s per image on the graphics processing unit (GPU), 640 × 480 pixels).

In 2015, Girshick et al. proposed the fast-RCNN detector based on their previous work. The main achievement of fast-RCNN is that it realizes a multi-task learning method which simultaneously trains the target classification network and bounding box regression network while network fine-tuning. On the VOC2007 dataset, fast RCNN achieves the mAP of 70% compared with 58.5% achieved by RCNN. Because external algorithms are still needed to extract the target candidate box in advance, they cannot achieve end-to-end processing.

Faster RCNN is an end-to-end deep learning detection algorithm with fast processing speed (17 FPS, 640 × 480 images). The main innovation of faster RCNN is that it proposes the region proposal network (RPN) and designs a “multi-reference window” to combine external object proposal detection algorithms (such as selective search or edge boxes) to the same deep network. From R-CNN to faster-RCNN, candidate region generation, feature extraction, candidate target validation, and bounding box regression tasks are gradually unified into one framework. The detection accuracy is increased from 58.8% achieved by RCNN to 78.8% and the detection speed is also increased.

In the specific domain of small object detection, such as vehicle detection in aerial images, the algorithms mentioned above are not applicable because the vehicles in these images have special characteristics e.g., small size, low resolution, and inconspicuous features. Small object detection is still one of the problems to be overcome urgently by computer vision. In other words, more pertinent networks should be designed for small object detection. Although some datasets have been used for small object detection, the number of samples in these datasets are simply not comparable to that of the conventional datasets. For example, the ImageNet [18] dataset contains 1,034,908 images with bounding box annotations, while a specially-made small object dataset (Vehicle Detection in Aerial Imagery, VEDAI) has only 1210 images [19]. This also brings challenges to the task of small object detection, the performance of the detector should be improved on the basis of a small amount of training samples. In the following, some specially designed object detection algorithms in aerial images will be systematically introduced.

In 2015, Razakarivony S et al. put forward VEDAI (a new database of aerial images [19]). They compared several object detection algorithms and found that most of the algorithms are not suitable for small object detection.

In 2017, the Lawrence Livermore National Laboratory of the United States [1] proposed an algorithm which modifies faster-RCNN to train the model for positioning small vehicles in VEDAI. The algorithm modified the anchors used in the RPN module of faster-RCNN and adjusted the input of RPN. The experiments showed that the modified faster-RCNN had substantial improvements in mAP, compared to the template-based sliding window methods.

In 2018, Yohei Koga et al. applied hard example mining (HEM) to the training process of a convolutional neural network for vehicle detection in aerial images [2]. Yohei Koga et al. used a sliding window method and CNN architecture. Candidate bounding boxes were scattered densely over an entire image and then those with no existence of vehicles were screened out. HEM was applied to the training of CNN used for the screening. The proposed method successfully promoted learning finer features and improved accuracy.

Yang et al. proposed a novel double focal loss convolutional neural network framework (DFL-CNN) in 2018, which was also an improved version of faster-RCNN [20]. DFL-CNN used skip connection to combine the features (conv5-3 and conv5-5) of faster-RCNN, which can enhance the network’s ability to distinguish individual vehicles in a crowded scene. To address the challenges of imbalance between each class and between easy/hard examples, it adopts focal loss function instead of cross-entropy function in both of the region proposal stage and the classification stage. The proposed network outperforms many others.

Ding et al. proposed a region of interest (RoI) transformer to solve the mismatches between the Region of Interests and objects [21]. These mismatches can be found when small objects are packed densely in aerial images. The experimental results demonstrated that by utilizing a rotated position sensitive RoI transformer based on a rotated RoI learner, the proposed algorithms can achieve a better performance than the deformable position sensitive RoI pooling method.

Low-level features of FPN [22] correspond to large targets, while the path between high-level features and low-level features is long, which increases the difficulty of accessing accurate positioning information. In order to shorten the information path and enhance the feature pyramid with low-level accurate positioning information, PANet [22] creates bottom-up path enhancement based on FPN, thus improving the ability to detect small objects.

In 2019, Cheng et al. proposed a CNN model based on rotation invariant and Fisher’s discrimination [23]. This model proposes an objective function, which can be optimized to carry out rotation invariant constraints and fisher discrimination on the generated CNN features. Hu et al. focuses on the large variance of scales, and designs a scale-insensitive convolution neural network which accomplishes by a context-aware RoI pooling and a multi-branch decision network [24]. Ju et al. proposed a specially designed network for small object detection [25]. This network combines ‘dilated module’ with feature fusion and ‘pass-through module’, and performs at the same level with YOLO V3 with much higher processing speed. Mandal et al. also designed a fast small object detector, and named it SSSDET (simple short and shallow network) [26]. According to their test, this algorithm outperforms YOLO V3, but it can be processed at the same speed as YOLO V3-Tiny. A new airborne image dataset named ABD was also proposed in this paper.

In 2020, Feng et al. focus on vehicle trajectory data under mixed traffic conditions [27]. Through detecting vehicles from UAV videos under mixed traffic conditions, a novel framework for accurate vehicle trajectory construction is designed. Zhou et al. focus on detecting vehicle when the vehicle logo has motion blur, and design a Filter-DeblurGAN which possesses, a judgment mechanism, to judge whether the image needs to be deblurred [28]. Moreover, a new vehicle logo dataset named LOGO-17 was released. Mandal et al. proposed a one-stage vehicle detection network named AVDNet. The proposed algorithm adopts specially designed ConvRes residual Blocks and enlarged output feature maps. According to the experiments, the proposed algorithm outperforms YOLO V3, faster R-CNN, and RetinaNet. Liao et al. were aware of the problem of mismatching when detecting dense and small objects in aerial images, and designed a local-aware region convolutional neural network (LRCNN) to solve this problem [29]. Rabbi et al. designed an edge-enhanced super-resolution GAN (EESRGAN) to enhance the quality of remote-sensing images [30]. The experimental results demonstrate that EESRGAN and Edge-Enhanced network can improve the performance of some object detectors, e.g., faster-CNN and single-shot multibox detector.

## 3. Method

**Figure 1:**
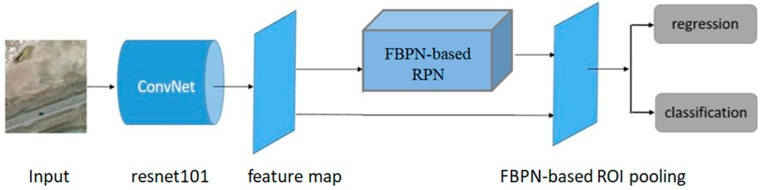
Overview of the proposed framework.

The framework is shown in Figure 1. It is improved based on faster RCNN. In this framework, the FBPN is proposed and combined with faster RCNN for better feature extraction and expression.

### 3.1. Introduction of Proposed Framework

Faster RCNN is one of the state-of-the-art detection frameworks, its simplicity and robustness have attracted a lot of researchers since 2015. It has been proven that faster R-CNN achieves the good detection accuracy on various datasets, such as PASCAL VOC 2007, 2012, and MS COCO [9]. Instead of selective search used in [8], faster RCNN utilizes a RPN for generating high-quality region proposals. Then these region proposals are fed upstream into the regression network and classification network. In the proposed framework, FBPN is adopted to faster RCNN because it can combine features from shallow layers and deep layers which are suitable for vehicle detection in aerial images. It can be seen from Figure 1, the FBPN-based RPN and FBPN-based RoI pooling are two important parts of the proposed framework. FBPN-based RPN is designed to generate region proposals and FBPN-based RoI pooling is used to extract features. Resnet-101 model [3] is selected as the backbone network instead of the VGG-16 model. Many papers have revealed that the network depth is important for performance improvement, and some nontrivial visual detection tasks have also greatly benefited from very deep models [23,24]. According to our experiments, Resnet-101 model performs better than VGG-16 in aerial images detection.

### 3.2. Introduction of Feature Balanced Pyramid Network (FBPN)

Recognizing objects of different sizes is a fundamental challenge in computer vision. A traditional algorithm like “Pyramid methods in image processing” [17] uses an original image to construct image pyramid and obtains features of different scales from image pyramid. However, features based on this method are computed on each of the image scales independently, which is computation intensive. The improved algorithm [31] carries out convolutions and pooling operations on the original image to obtain feature maps of different sizes. The experiments show that feature maps from high layers provide more semantic information, which can help us detect the targets. Many deep networks (VGG, ResNet, Inception) utilize this method to make predictions. However, its disadvantage is that only the features of the last layer in the deep network have been used, and the features of other layers have all been ignored. The feature pyramid network can combine features from the shallow layers with those from deep layers to improve the detection accuracy. However, it makes integrated features focus more on adjacent resolution but less on others, and the semantic information contained in non-adjacent levels would be diluted once perfusion during the information flow.

**Figure 2:**
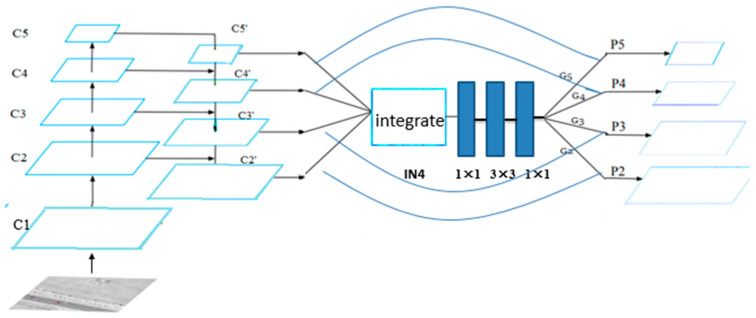
The structure of feature-balanced pyramid network (FBPN).

In order to solve this problem, our framework combines two types of features. As is shown in Figure 2, the bottom-up pathway is the feed-forward computation process of Resnet-101. The feature activations outputs are used by each stage’s last residual block (conv1, conv2_x, conv3_x, conv4_x, conv5_x) and the outputs of these last residual blocks are donated as {C1, C2, C3, C4, C5}. C1 is not included in the pyramid network because of its large memory footprint. The top-down pathway upsamples higher feature maps and then connect the features to the previous layer via lateral connections. For example, C3′ is upsampled by a factor of 2. The upsampled map is then merged with the bottom-up map C2 (which undergoes a 1 × 1 convolutional layer to reduce channel dimensions) by element-wise to generate C2′. The final set of feature maps is {C2′, C3′, C4′, C5′}.

To integrate multi-level features and preserve their semantic hierarchy at the same time, we first resize the size of multi-level features {C2′, C3′, C5′} to C4′, with interpolation and max-pooling respectively. Once the features are rescaled, the balanced semantic feature ‘IN4′ is obtained in two ways. One is the simple average, and the formula is as follows:

IN4 = (C2′ + C3′ + C4′ + C5′)/4

Another way is that {C2′, C3′, C4′, C5′} are concatenated/integrated on the depth first, then these features are processed by 1×1 convolution kernels to reduce dimensions. We have compared the two methods and found that the second method works better.

**Figure 3:**
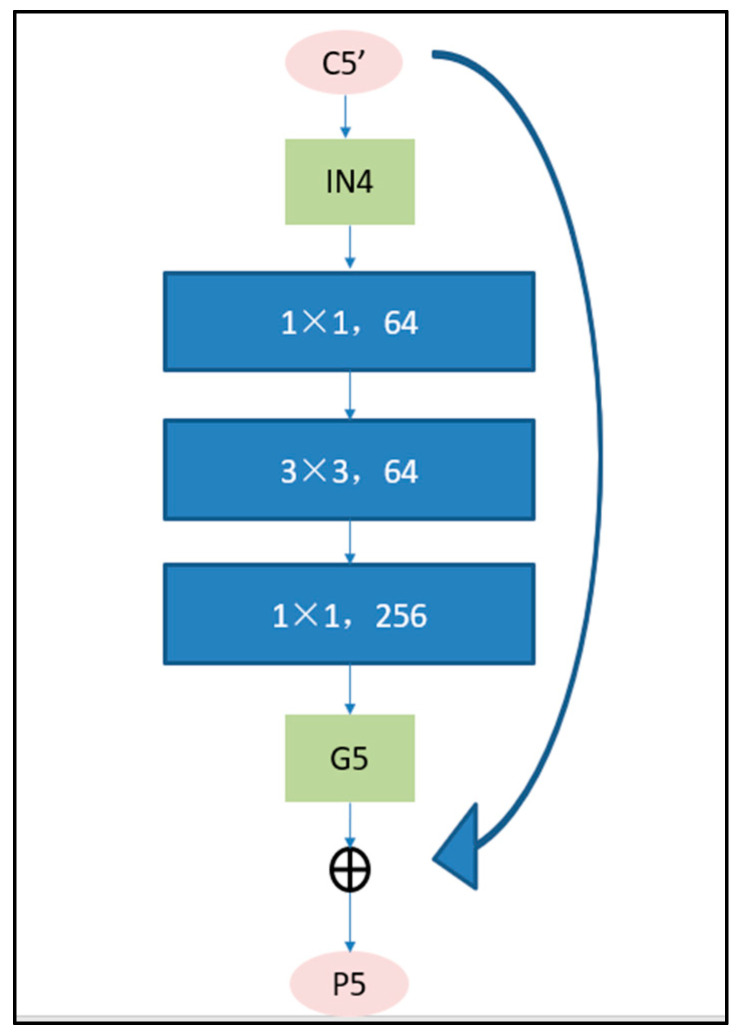
The progress of feature enhancement.

The balanced feature IN4 is then rescaled using the same but reverse procedure to strengthen the original features. IN4 can be further refined to get better feature expression using the 1 × 1, 3 × 3, 1 × 1 convolutions. With this method, features from low-levels to high-levels are aggregated at the same time. The aggregated features are {G2, G3, G4, G5}. The final feature P is the combination of G and C. For example, P5 is the combination of C5 and G5. The detailed process is shown in Figure 3. The outputs {P2, P3, P4, P5} are used for object detection following the same pipeline in FPN.

### 3.3. The Combination of FBPN and Faster Region Convolutional Neural Network (RCNN)

FBPN is rather than an object detection network, therefore, it is applied in two main aspects of the proposed framework: RPN and fast RCNN to extract more effective features.

#### 3.3.1. FBPN-Based Region Proposal Network (RPN)

RPN is a fully convolutional network that simultaneously predicts object bounding boxes and scores at each position. The structure of the RPN can be seen in [9]. To generate region proposals, an RPN is constructed on top of the activation map of the last shared convolutional layer. The input of RPN is an N × N (typically 3 × 3) spatial window of the input convolutional feature map. The outputs of this convolutional layer’s sliding window are then mapped to a low-dimensional (e.g., 256) feature vector [1]. Finally, these low-dim features are fed into two fully connected layers: bounding box regression layer (reg layer) and classification layer (cls layer). The detailed explanations about RPN can be found in [9].

The default anchor scales of faster RCNN are $\left\{128^{2},256^{2},512^{2}\right\}$ pixels, and the default aspect ratios are {1:2,1:1,2:1}, which cater to both the larger sizes of objects and more dramatic scale variances. For aerial image datasets such as VEDAI that contain targets on the order of tens of pixels, the scales used in faster RCNN are inadequate. In the FBPN-based RPN module, each pyramid level uses a single scale’s anchor. {P2, P3, P4, P5} have anchors of $\left\{32^{2},64^{2},128^{2},256^{2}\right\}$. Considering the diversity of vehicle objects, the anchor ratio is reset to {0.5,1.5/2,1,2.5/2,2}.

#### 3.3.2. FBPN-Based Region of Interest (RoI) Pooling

The RoI pooling process uses the pooling method to turn the RoIs of different sizes in the input feature map into fixed-size output feature maps. It has two inputs: feature map generated by Resnet-101 and the outputs of RPN. The features from different pyramid levels are used as the input of RoI pooling layer for RoIs with different scales. For large-scale RoIs, the later pyramid level, such as P5, is adopted. For small RoIs, (P4, P3) are used. In order to assign RoIs of different scales to the pyramid levels, feature pyramid network uses a coefficient *k* [31]. *k* is also redefined in the proposed framework, which is more suitable for the small vehicle detection in aerial images. In the proposed framework, the *k* is formally redefined as:

(1) $$k=\left\lfloor k_{0}+\log_{2}(\sqrt{wh}/1024)\right\rfloor$$

where *k_0_* = 4, *w* and *h* are the length and width of the RoI region.

#### 3.3.3. Focal Loss Function

Cross-entropy loss function describing the distance between two probability distributions is the most popular loss function used for object detection. When the cross-entropy loss function is smaller, two probability distributions are more similar. It can improve the imbalance between positive and negative samples to a certain extent. However, as for the imbalance between easy and hard examples, it does not perform well. To solve this imbalance, some hard samples mining strategy should be designed. In this paper, focal loss function [31] is adopted in the region proposal stage. The focal loss function is an optimization of the cross-entropy loss function and the details can be found in the paper [32]. The original loss function of region proposal stage is formally defined as:

(2) $$L_{\mathrm{rpn}}=\frac{1}{N_{cls}}\sum L_{cls}\left(p_{i},p_{i}^{*}\right)+\lambda \frac{1}{N_{reg}}\sum p_{i}^{*}L_{smo},$$

(3) $$L_{smo}\left(i\right)=\left\{ \begin{array}{ll} 0.5i^{2} & if|i|<1\\ |i|-0.5 & \mathrm{otherwise} \end{array}\right.,$$

(4) $$L_{cls}=-\log\left(p_{t}\right),p_{t}=\left\{ \begin{array}{ll} p & if\;y=1\\ 1-p & \mathrm{otherwise} \end{array}\right.,\;y\in \{-1,+1\}$$

where $L_{cls}$ is the conventional cross-entropy and $L_{smo}$ is the smooth loss function. $N_{cls}$ and $N_{reg}$ denote the total number of samples and the total number of positive samples respectively. In the proposed framework, the $L_{cls}$ is changed as follows:

(5) $$L_{cls}=-\alpha {\left(1-p_{t}\right)}^{\gamma }\log\left(p_{t}\right)$$

A couple of parameters (i.e., α,γ) are tested in the evaluation process and the best is α=0.3 ,γ=2.

## 4. Experiments and Results

### 4.1. Dataset

#### 4.1.1. Brief Introduction

There are many conventional target detection datasets, such as MS COCO, PASCAL VOC, ImageNet and so on. Many frontier object detection algorithms (faster RCNN, Yolo, SSD, mask RCNN, etc.) are trained and evaluated on these datasets. However, the detectors trained on conventional datasets do not perform well on aerial images because of the following reasons. First, aerial images are generally viewed from high altitude, but most of the conventional datasets are from the ground-level perspective. As a result, the features used in detectors that are well trained on conventional datasets may be ineffective in aerial image detection. Second, many objects in aerial images are very small (tens or even fewer pixels), which results in the lack of effective information compared with objects in ground view images. The detection algorithms based on CNN perform well on conventional target detection datasets. But for small objects, the pooling layers of CNN will further reduce information. For example, a 24 × 24 object may have only one pixel after four levels of pooling, which makes the object difficult to distinguish. Last but not least, aerial images have a large field of vision (usually one aerial image covers several square kilometers.) which may contain a variety of background that cause strong interference to the detection process. Based on the reasons above, some datasets such as the DLR Munich vehicle dataset [33], the Overhead Imagery Research Data Set [34], VEDAI [1] and UCAS-AOD [35] have been proposed and used for vehicle detection in aerial images. Experiments in this paper are performed both on VEDAI, UCAS-AOD, and DOTA [36] datasets.

#### 4.1.2. VEDIA, USCAS-AOD and DOTA Datasets

The VEDAI dataset contains 9 classes of objects, including cars, pickups, trucks, ships, tractors, camping cars, vans, vehicles and planes. In this paper, our attention is focused on small traffic vehicles, namely the cars, pickups, tractors, camping cars, trucks and vans classes. A total of 3090 instances across these six classes are presented in 1089 images of the VEDAI dataset; 80% of the images are randomly chosen for the training process and the remaining 20% for the testing process.

**Figure 4:**
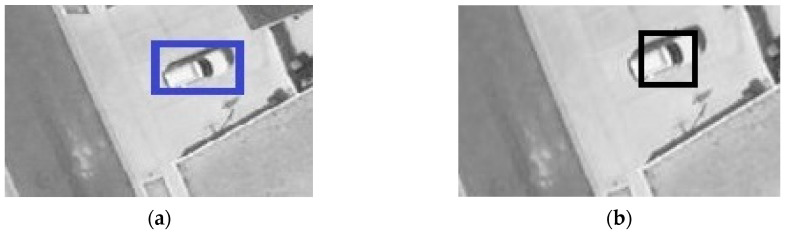
Two annotation methods: (**a**) sample of annotation 1. (**b**) sample of annotation 2.

The original annotation of VEDAI dataset includes the centroid, orientation, and coordinates of four corners of each instance. Annotation 1 [1] retains the centroids and generate 40 × 40 pixel square bounding box around the centroids for the 1024 × 1024 resolution imagery (20 × 20 pixel square bounding box around the centroids for 512 × 512 resolution imagery). In this paper, annotation 2 compares the x, y coordinates of the four corners and selects the Xmin, Ymin, Xmax, Ymax. The detailed samples can be seen in Figure 4.

**Figure 5:**
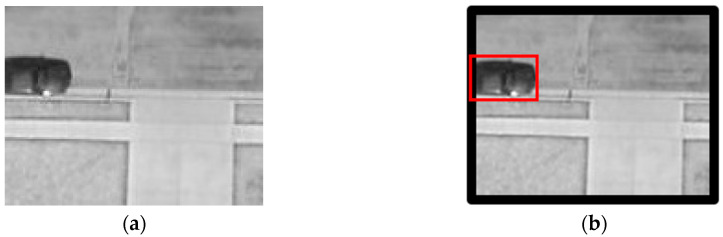
(**a**) Original image. (**b**) Padding image.

By observing a large number of dataset labels, it has been found that many vehicles are at the edge of the images, which will lead to unavoidable overflow errors. For each image in VEDAI, padding has been added to solve the problem (Figure 5).

UCAS-AOD (Dataset of Object Detection in Aerial Images) dataset is annotated by the pattern recognition laboratory in University of Science and Technology of China. The dataset contains automobile, aircraft, and background negative samples. In this paper, all the automobile images in UCAS-AOD dataset are used in the training or evaluating process.

As a large-scale dataset, the DOTA dataset is composed of 2806 aerial images with various resolutions, and these images contain different objects from 15 various categories. Comparing to normal vehicles, the objects in some categories are large, e.g., basketball court, soccer-ball field, swimming pool, bridge, and harbor. To address the difficulties of detecting small vehicles, in this section, only the objects annotated as ‘small vehicles’ are used in the experiments. Since the original DOTA images can be as large as 4000 × 4000, they are cropped into smaller images with a resolution of 600 × 600 and a stride of 520 pixels.

### 4.2. Evaluation Method

As for the performance evaluation of the proposed model, the standard precision (*P*), recall (*R*) and average precision (AP) are used. The F1-score is also used in our evaluation system as an important reference index. The definitions of these metrics are formally described as:

(6) $$\mathrm{Recall}\;\mathrm{Rate}\;(R)\;=\;\frac{TP}{TP+FN}$$

(7) $$\mathrm{Precision}\;\mathrm{Rate}\;(P)\;=\;\frac{TP}{TP+FP}$$

(8) $$F1\_score=\frac{2\times R\times P}{R+P}$$

where *TP*, *FN*, *FP* denote the true positive, false negative and false positive respectively. The definition of what is a positive detection is the standard intersection over union (IoU) criterion adopted by the Pascal VOC or MS COCO [20]. The detections with IoU value greater than 0.5 is defined as true, otherwise, it is false.

### 4.3. Training Details of Proposed Framework

To train the proposed framework, stochastic gradient descent is applied with weight decay of 0.0001 and momentum of 0.9. There are 70 k iterations in total during the whole training process. The learning rate is set to 0.001 for the first 30 k iterations and 0.00001 for the following 40 k iterations.

### 4.4. Results on VEDAI Dataset

The evaluation results of the proposed frameworks and other published algorithms on VEDIA are shown in Table 1. The detailed results of the proposed frameworks, including recall, precision, F1-score, mAP and process time per image, can be found in Table 2.

Firstly, compared with conventional faster RCNN(with an mAP of 70.9%), the improved faster RCNN [37] reaches an mAP of 74.3%, which is a solid improvement. The improved faster RCNN uses ResNet-101 instead of VGG-16, and the annotation method is also changed from Annotation1 to Annotation2. The initial learning rate and momentum value are set as 0.001 and 0.9 respectively. Instead of the original ResNet bottleneck block used in improved faster RCNN, faster RCNN + Res2Net [38] utilizes the Res2Net module which is designed to represent features in multiple scales. The rest of these algorithms are kept the same. Thanks to the Res2Net module, comparing to improved faster RCNN, faster RCNN + Res2Net achieves a considerable 7.66% increment of mAP. The Waterfall [39] module is also evaluated in this experiment. Comparing to the ResNet and Res2Net block, a single Waterfall module contains more trainable parameters. As a result, the backbone structure used in the faster RCNN + Waterfall algorithm is changed to ResNet50 to keep the number of parameters to the same level of improved faster RCNN and faster RCNN + Res2Net. The dilation rates used in Waterfall module is changed from (6, 12, 18, 24) to (1, 2, 3, 4) to achieve better performance on small objects. Compared to the improved faster RCNN, faster RCNN + Waterfall also achieves 3% increment of mAP. Comparing the results obtained from faster RCNN, improved faster RCNN, faster RCNN + Res2Net, faster RCNN + WaterFall, it can be seen that although the latest residual blocks, e.g., Res2Net and Waterfall, introduce a certain level of multiple scales representations to the network, it cannot prevent the loss of low-level features that are critical for small object detection algorithms.

Secondly, the proposed framework 1.1 reaches an mAP of 88.2%. Compared with improved faster RCNN which achieves an mAP of 74.30, it can be seen that the proposed feature fusion module leads to a significant performance boost (13.9%). The structure of the proposed framework 1.1 is shown in Figure 1 (the backbone is Resnet101) and the training details are reported in Section 4.3. The proposed framework 1.1 uses the cross-entropy loss function.

Thirdly, compared with proposed framework 1.1, the framework 1.2 changes the loss function (using focal loss function instead of cross-entropy loss function). It reports an mAP of 88.82% (0.62% improvement compared with framework 1.1).

Fourthly, compared with proposed framework 1.2, framework 1.3 utilizes the images of two datasets (UCAS-ADO dataset and VEDAI dataset) during the training process and achieves an mAP of 91.27% (2.45% improvement compared with framework 1.2).

**Figure 6:**
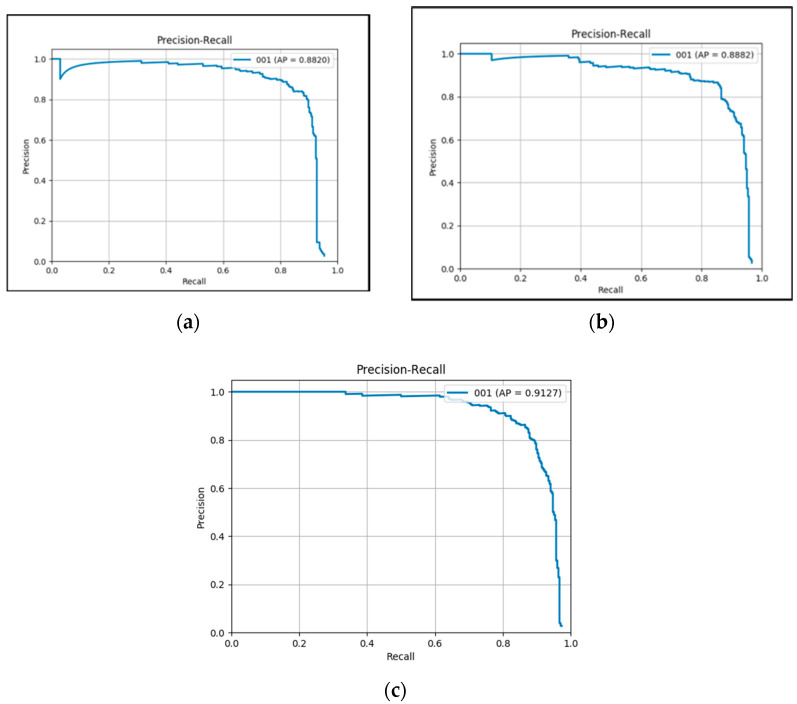
(**a**) P-R curve of framework1.1 (**b**) P-R curve of framework1.2 (**c**) P-R curve of framework 1.3.

Figure 6a–c show the P-R curve of the proposed frameworks.

**Table 1:** Comparison between proposed framework and other methods on VEDAI.

Method	mAP
AVDNet 2019 [40]	51.95
VDN 2017 [41]	54.6
DPM 2015 [32]	60.5
R^3−^Net (R + F) 2019 [42]	69.0
Faster-RCNN 2017 [32]	70.9
Improved Faster RCNN 2017 [37]	74.30
Ju, et al. 2019 [25]	80.16
YOLOv3_Joint-SRVDNet 2020 [43]	80.4
Faster RER-CNN 2018 [32]	83.5
YOLOv3_HR [43]	85.66
DFL 2018 [40]	90.54
Faster RCNN + Res2Net (resnet101) 2019 [38]	81.96
Faster RCNN + WaterFall (resnet50) 2019 [39]	77.36
Framework 1.1	88.20
Framework 1.2	88.82
Framework 1.3	91.27

**Table 2:** R, P, mAP, F1-score and test time on VEDAI dataset.

Framework	R	P	mAP	F1-Score	Test Time (Per Pic)
Improved Faster RCNN	80.7	63.1	74.3	70.8	0.048 s
Framework 1.1	84.5	85.6	88.20	85.9	0.049 s
Framework 1.2	85.7	86.7	88.82	86.5	0.049 s
Framework 1.3	86.5	87.5	91.27	87	0.049 s

**Figure 7:**
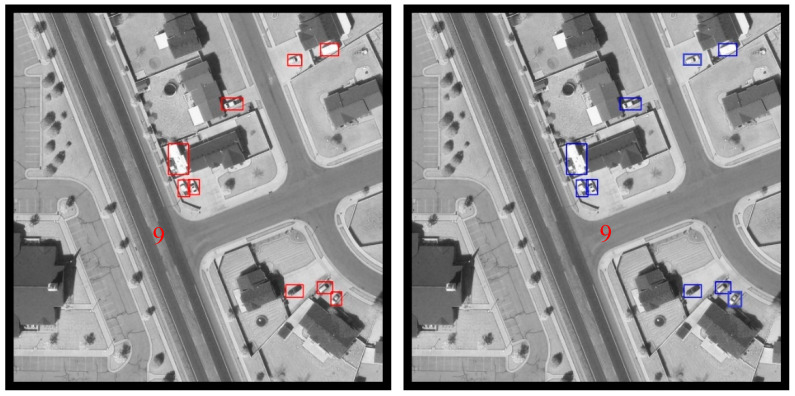

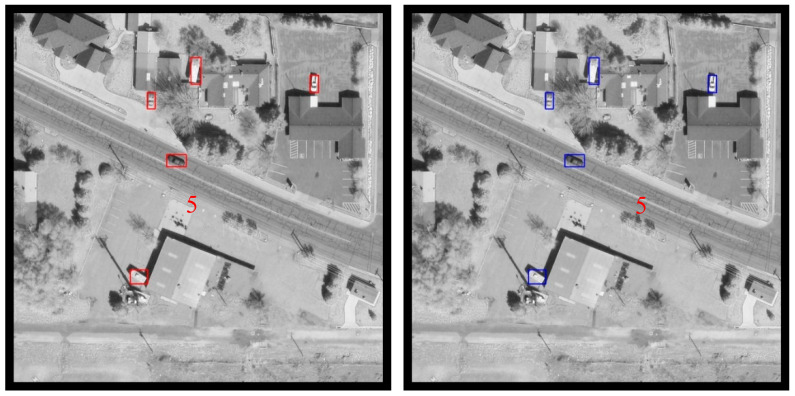
Detection samples from framework1.1 The left column contains the ground truth. The right column contains the results generated by framework1.1.

Samples of framwork1.1 are shown in Figure 7. The number on each image indicates the number of vehicles contained in that image. The left column contains the ground truth of the original picture which is annotated in the required form. The right column contains the results of our detecting algorithm. It can be seen that the proposed detection framework performs well on the VEDAI dataset.

**Figure 8:**
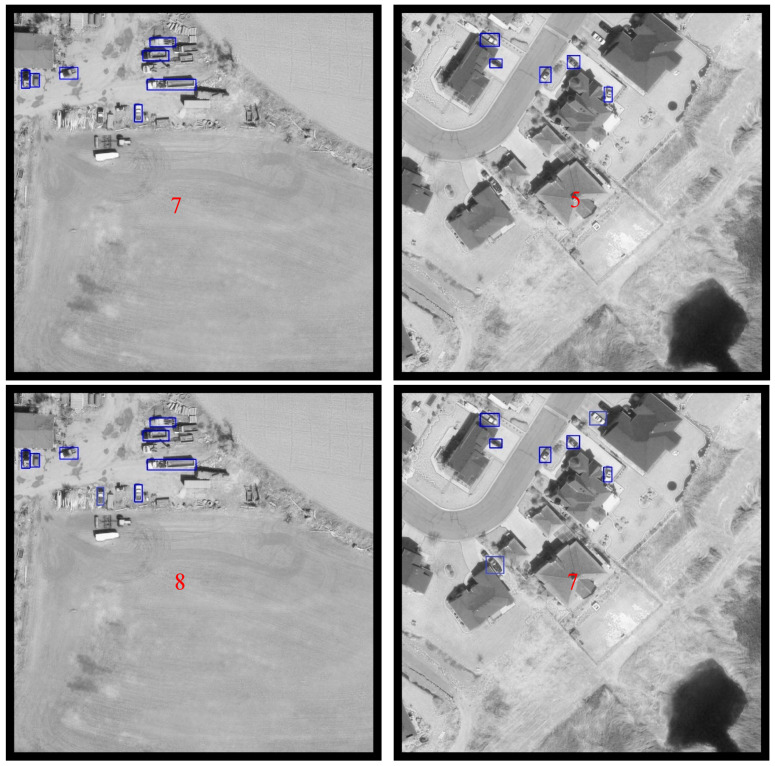

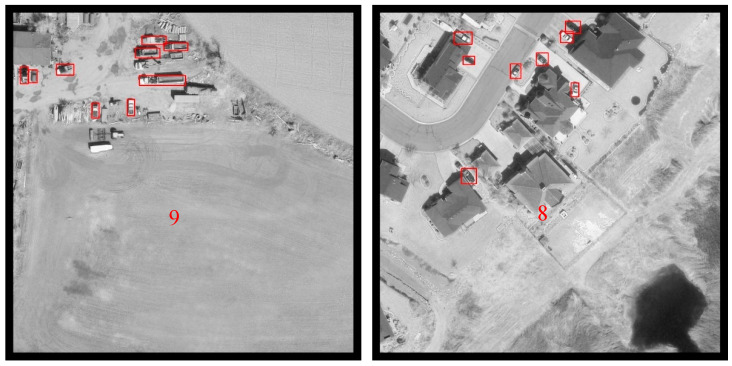
Comparisons between cross-entropy loss function and focal loss function. The first row contains results generated using cross-entropy loss function. The second row contains the results generated using focal loss function. The last row contains the ground truth.

The results comparison between the focal loss function and the cross-entropy loss function can be found in Figure 8. The first row contains the results generated using the cross-entropy loss function, and the second row contains the results generated using focal loss function. The last row contains the ground truth. Compared to the algorithm using cross-entropy loss function, the algorithm using focal loss function can detect more ‘true positive’ in the same figure.

**Figure 9:**
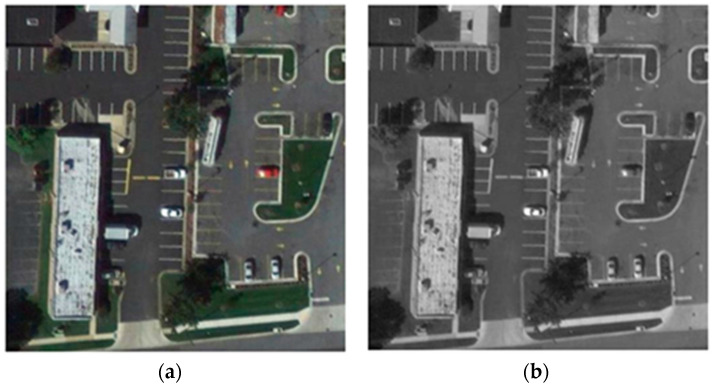
(**a**) The original image sample. (**b**) The processed image sample.

UCAS-AOD dataset is used in the experiments of this section for image augmentation (defined as framework 1.3). The UCAS-AOD dataset contains colour images provided from Google Earth and the size of vehicles is similar to the vehicle sizes in VEDAI dataset. In order to reduce the impact between these two datasets, the automobile images in UCAS-AOD dataset have been converted to grayscale image (details can been seen in Figure 9). In the experiments, the training dataset contains both UCAS-AOD images and the VEDAI training data. However, evaluating the dataset is randomly selected from VEDAI data only.

### 4.5. Results on UCAS-AOD Dataset

In the experiments of this section, the training dataset contains images from both VEDIA and UCAS-AOD datasets. However, the evaluation dataset is randomly chosen from UCAS-AOD dataset only. The comparative performance of the proposed framework (1.2) and other 16 state-of-the-art algorithms in terms of mAP based on UCAS-AOD is shown in Table 3. It can be seen that the proposed framework achieves an mAP of 96.18 which is better than other state-of-the-art algorithms. The much more complicated UCAS + NWPU +VS-GANs achieves the second place with an mAP of 96.12 by adding 2000 screened vehicle samples.

Table 4 shows the detailed evaluation results (i.e., recall(R), precision (P), mAP and F1-score) of framework 1.2 on UCAS-AOD dataset.

**Figure 10:**
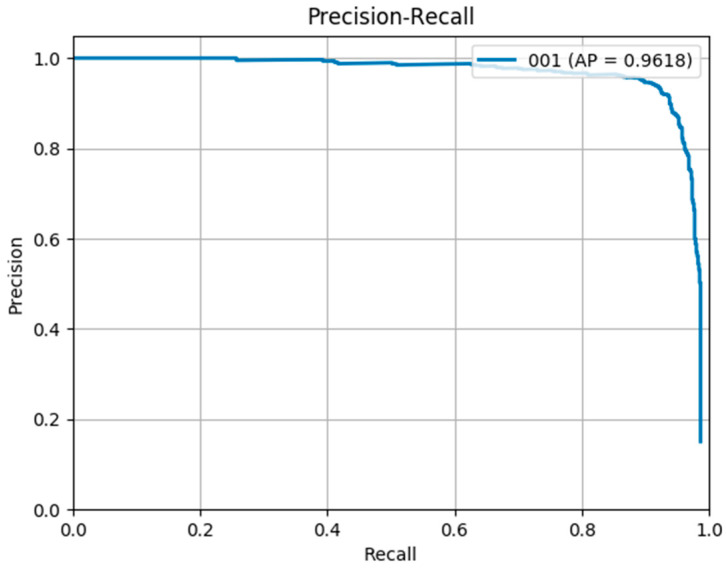
P-R curve of Framework 1.2 on UCAS-AOD.

Figure 10 demonstrates the P-R curve of Framework 1.2 based on UCAS-AOD dataset.

**Figure 11:**
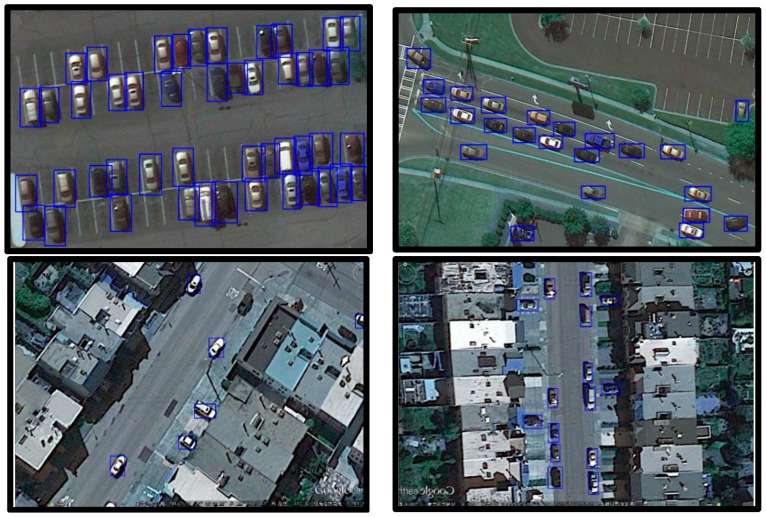
Detection samples on UCAS-AOD dataset using framework 1.2.

Figure 11 displays some samples generated by Framework 1.2 using images in UCAS-AOD dataset.

**Table 3:** Comparison between proposed framework and other methods on UCAS-AOD.

Method	mAP
YOLO v2 2017 [44]	79.20
SSD 2020 [12]	81.37
R-DFPN 2018 [45]	82.50
DRBox 2017 [46]	85.00
O^2−^DNet 2016 [47]	86.72
P-RSDet 2020 [48]	87.36
RFCN 2016 [49]	89.30
Deformable R-FCN 2017 [50]	91.7
S^2^ARN 2019 [51]	92.20
FADet 2019 [52]	92.72
RetinaNet-H 2019 [53]	93.60
R^3^Det 2019 [53]	94.14
A^2^RMNet 2019 [54]	94.65
SCRDet + + 2020 [55]	94.97
ICN 2018 [33]	95.67
UCAS + NWPU + VS-GANs 2019 [56]	96.12
Framework 1.2	96.18

**Table 4:** R, P, mAP, F1-socre of proposed Framework1.2 on UCAS-AOD dataset.

Framework	R	P	mAP	F1-Score
Framework 1.2	93.3	92.5	0.9618	92.89

### 4.6. Results on DOTA Dataset

In the experiments of this section, the training and evaluation images are from the DOTA dataset only. All the objects annotated as ‘small vehicle’ are used in this section. Table 5 contains comparison results between the proposed framework (1.2) and other 12 state-of-the-art algorithms evaluated based on images containing objects from ‘small-vehicle’ category only in DOTA dataset. It can be seen that the proposed framework performs better than other state-of-the-art algorithms. It reaches an mAP of 88.76%, which is improved compared with Ju, et al. [25] (88.63%). Please note that Table 5 also contains results from three other algorithms that with a star notation in front of them. These algorithms are evaluated by objects not only from ‘small-vehicle’ category. The evaluation result of these algorithms cannot compare directly with results from other algorithms. But it demonstrates that when evaluating algorithms using the DOTA dataset, the choice of categories used in the evaluation process can affect the evaluation result significantly. By adding large objects into the evaluation dataset, the standard YOLO V3 can achieve outstanding performance.

Table 6 shows the detailed evaluation results (i.e., recall (R), precision (P), mAP and F1-score) of framework 1.2 on DOTA dataset.

**Figure 12:**
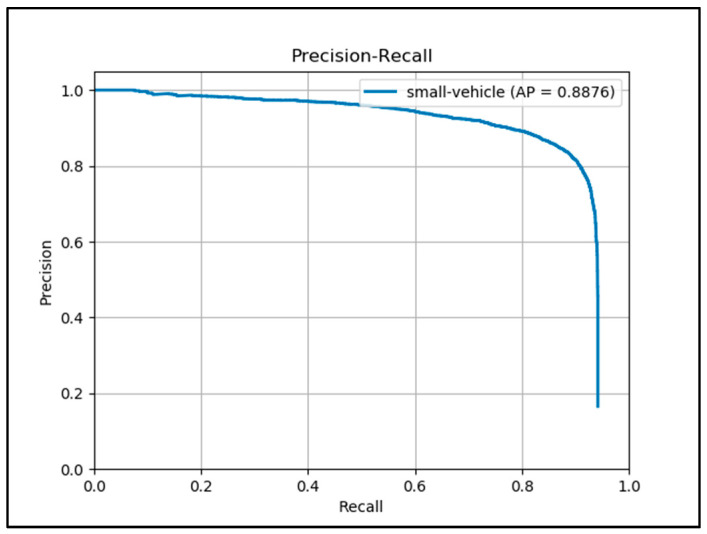
P-R curve of Framework 1.2 on DOTA.

Figure 12 demonstrates the P-R curve of Framework 1.2 based on DOTA dataset.

**Figure 13:**
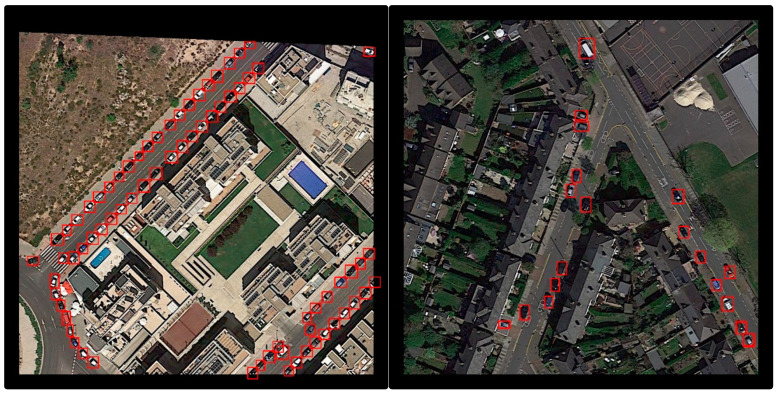

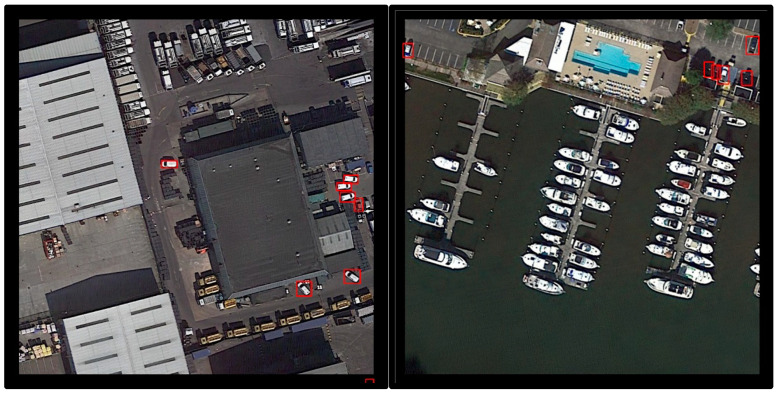
Detection samples on DOTA dataset using framework 1.2.

Figure 13 displays some samples generated by Framework 1.2 using images in DOTA dataset.

**Table 5:** Comparison between proposed framework and other methods on DOTA.

Method	mAP(Small Vehicle)
DFL 2018 [40]	45.56
Yang et al. 2018 [57]	61.16
Ding, et al. 2018 [21]	70.15
Faster RCNN Adapted 2018 [58]	74.9
DYOLO Module B 2018 [58]	76.0
SSD Adapted2018 [58]	76.3
DFRCNN 2018 [59]	76.5
* SSSDet 2019 [26]	77.22
L-RCNN 2020 [29]	77.86
DSSD 2017 [60]	79.0
DYOLO Module A 2018 [58]	79.2
* AVDNet 2019 [40]	79.65
RefineDet 2018 [58]	80.0
* YOLO v3 2019 [26]	88.31
Ju, et al. 2019 [25]	88.63
Framework 1.2	88.76

* These three algorithms are evaluated by objects not only from ‘small-vehicle’ category. The evaluation result of these algorithms cannot compare directly with results from other algorithms.

**Table 6:** R, P, mAP, F1-socre of proposed Framework1.2 on DOTA dataset.

Framework	R	P	mAP	F1-Score
Framework 1.2	84.5	87.3	88.76	85.9

## 5. Conclusions

In this paper, a specialized framework is proposed in order to improve the performance of vehicle detection in aerial images. Unlike the other state-of-the-art detection models, the proposed detector combines the feature-balanced pyramid network (FBPN) with faster RCNN. The features extracted by FPN pay more attention to the influence between adjacent levels. FBPN can combine features of adjacent and non-adjacent levels and makes the whole detection framework perform better. In the FBPN framework, the improved FBPN-based RPN and FBPN-based RoI pooling are two important parts. The former uses a single scale anchor with various ratios in each pyramid level for generating more diverse anchors that are suitable for small vehicle detection. The latter adjusts the input and selectively uses fused features for extracting more effective features. The backbone of faster RCNN is replaced by Resnet-101 because it is more suitable for the proposed framework according to the experiments. In order to improve the imbalance between easy and hard examples, the focal loss function is used instead of the conventional cross-entropy loss function. The proposed framework is trained and evaluated using VEDAI, UCAS-AOD, and DOTA datasets. The experimental results show that the proposed framework outperforms other state-of-the-art algorithms.
